# Hydrolysates of Fish Skin Collagen: An Opportunity for Valorizing Fish Industry Byproducts

**DOI:** 10.3390/md15050131

**Published:** 2017-05-05

**Authors:** María Blanco, José Antonio Vázquez, Ricardo I. Pérez-Martín, Carmen G. Sotelo

**Affiliations:** Instituto de Investigaciones Marinas (IIM-CSIC), Eduardo Cabello, 6, Vigo, Galicia 36208, Spain; jvazquez@iim.csic.es (J.A.V.); ricardo@iim.csic.es (R.I.P.-M.); carmen@iim.csic.es (C.G.S.)

**Keywords:** collagen, enzymatic hydrolysis, antioxidant activity, *β*-carotene, DPPH, ABTS

## Abstract

During fish processing operations, such as skinning and filleting, the removal of collagen-containing materials can account for up to 30% of the total fish byproducts. Collagen is the main structural protein in skin, representing up to 70% of dry weight depending on the species, age and season. It has a wide range of applications including cosmetic, pharmaceutical, food industry, and medical. In the present work, collagen was obtained by pepsin extraction from the skin of two species of teleost and two species of chondrychtyes with yields varying between 14.16% and 61.17%. The storage conditions of the skins appear to influence these collagen extractions yields. Pepsin soluble collagen (PSC) was enzymatically hydrolyzed and the resultant hydrolysates were ultrafiltrated and characterized. Electrophoretic patterns showed the typical composition of type I collagen, with denaturation temperatures ranged between 23 °C and 33 °C. In terms of antioxidant capacity, results revealed significant intraspecific differences between hydrolysates, retentate, and permeate fractions when using *β*-Carotene and DPPH methods and also showed interspecies differences between those fractions when using DPPH and ABTS methods. Under controlled conditions, PSC hydrolysates from *Prionace glauca*, *Scyliorhinus canicula*, *Xiphias gladius,* and *Thunnus albacares* provide a valuable source of peptides with antioxidant capacities constituting a feasible way to efficiently upgrade fish skin biomass.

## 1. Introduction

As the human population is growing and their consumption behavior changing, the worldwide demand for fishery products is increasing as is the demand for ready to cook meals in the form of loins or steaks. These kinds of processed products generate a large amount of by-products in the form of skin, bones, viscera, heads, scales, etc. Those organic materials are considered postharvest fish losses (by-products) and are a main concern for current fishery management policies because they represent a significant source of valuable compounds as proteins, fat, minerals, etc. Although part of these by-products are already being used, either for fish meal or oil production (35% of world fishmeal production was obtained from fish byproducts) [1]; this kind of utilization is considered to produce very little added-value, but due to present technological developments, a more valuable and profitable use is possible [2].

Fishing activity in Galicia (North-West Spain) constitutes a key sector for the economy of the region, with a high concentration of small, medium, and big businesses dedicated to fish processing activities that render a wide variety of by-products susceptible to valorization. During fish processing operations the removal of collagen-containing materials (mainly skin, bones and scales) could account for as much as 30% of the total by-products generated after filleting (75% of the total catch weight) [3,4].

Although collagen is the main protein component of fish skin and its particular heterotrimeric structure [*α*_1_(I)]_2_ *α*_2_(I) has been previously described, there have been only a few publications describing the properties of fish skin collagen hydrolysates [5,6,7], and even less research has been conducted on the characterization of hydrolysates obtained from pepsin soluble collagen of marine origin [7]. As acid solubilisation of collagen has been shown to render low yields, enzymatic proteolysis has been studied as an alternative to enhance the yield and at the same time obtaining hydrolysates with good nutritional composition, increased solubility and better emulsifying, foaming, and gelating properties, as well as biologically active peptides [8,9,10].

Two sharks, blue shark (*Prionace glauca*; PGLA) and small-spotted catshark (*Scyliorhinus canicula*; SCAN), and two bonny fishes, yellowfin tuna (*Thunnus albacares*; TALB) and swordfish (*Xiphias gladius*; XGLA) were selected since a significant amount of these are industrially processed generating significant amounts of skin [11,12,13]. The objective of this study was to evaluate the potential use of skins which are obtained as a by-product of the fish processing industry to obtain fish skin collagen hydrolysates and to test the influence of some biochemical properties, as the amino acid content or molecular weight, on antioxidant capacity of hydrolysates. This is the first time, as far as we know that the extraction, characterization and comparison of collagen hydrolysates from these species, is described.

## 2. Results and Discussion

Fish skin can be an important by-product for some fishery industries, for example some companies produce pieces of skinned and deboned fish which render important amounts of skins and bones as by-products. One of the problems associated with these by-products is the heterogeneity of them: they are originated from different species, previous frozen storage conditions can be different (frozen storage in brine), they can be mixed with bones or other by-products, etc. Appropriate management of these by-products should take into account these problems, and one important and initial step is to estimate the value associated with each type of product. Therefore, the initial chemical characterization and the estimations of collagen content are important data in evaluating the potential value of these by-products. Low yields of collagen extraction can be expected in industrial conditions because of the previous treatment and storage history of the raw materials. Hydrolysis would help to overcome some of the problems associated with these previous treatments, increasing the yield of a valuable product, collagen hydrolysates, which has many interesting properties, such as antioxidant activity [14,15].

### 2.1. Chemical Composition of Skin By-Products

#### 2.1.1. Proximate Composition

Table 1 shows the chemical composition of the skins of the four species analysed, these were similar to the skins of other fish species. Skin of the two elasmobranch contained similar amounts of protein, while swordfish skin presented the lowest protein content of all species, while those from tuna were the highest. In the case of swordfish, it is remarkably the highest lipid content (30.53%), which may also be the target of valorisation for this type of by-product. The higher ash content in the skin of the small-spotted catshark is remarkable and it could be attributed to its particular skin structure; a thinner skin with a higher proportion of scales compared to the skin of the blue shark. The skin of the blue shark is thicker and presents two different layers with scales only present in the upper layer.

#### 2.1.2. Hydroxyproline (HPro) Content

Hydroxyproline has been used as a method to quantify the amount of collagen in a particular tissue [16]. This analytical approach was used to estimate the collagen content in the skin of all the species analyzed, assuming that all HPro content of skin is due to collagen and taking into account that the ratio of HPro in collagen is 12.5 g of HPro/100 g of collagen [17]. Table 2 shows that the collagen content was higher in the skin of TALB, followed by the two species of elasmobranch which showed similar values (SCAN and PGLA), and finally the lowest value corresponded to the skin of XGLA, these results are in coherence with the protein content found in the skin of these species (Table 1). Collagen content reported previously for other fish species was similar with slight variations depending on the species [18].

Sotelo et al. [19] have reported a low collagen content in the skin of SCAN (11.6% in a wet basis), which may be explained by differences in the previous treatment of skins for this species (used fresh in this study).

### 2.2. Extraction of Collagen

#### 2.2.1. Yield of PSC

Previous reports have shown that pepsin enhances the extraction efficiency in collagen because it is able to cleave specifically telopeptide regions of collagen [20,21]. Besides, by hydrolysing the non-triple helice domain, non-collagen proteins are more easily removed, and thus collagen becomes readily solubilized in acid solution and the antigenicity caused by telopeptides is reduced, obtaining a collagen with higher purity with the possibility of using it in different applications [22,23,24].

Table 2 shows PSC yields obtained for PGLA, SCAN, TALB, and XGLA. Extraction yields obtained for PGLA and SCAN were similar to other PSC extracted from different fish species, such as bigeye snapper skin [25], brownstripe red snapper skin [26], or largefin longbarbel catfish [27]. However, the yields obtained for TALB and XGLA are lower than those values. While TALB showed the highest collagen content values (determined by means of hydroxyproline analysis in skin), it also showed (together with XGLA skins) lower extraction yields (PSC_1_ and PSC_2_). These results could be attributed to several factors such as differences in the structure of the collagen fibers or the storage conditions; processing of tuna usually involves freezing and frozen storage, most of the times in brine. This treatment may cause protein denaturation, a higher degree of crosslinking and therefore lower collagen solubility and extraction yields [27,28,29,30].

#### 2.2.2. Characterization of PSC

##### Polyacrylamide Gel Electrophoresis (SDS-PAGE)

**Figure 1:**
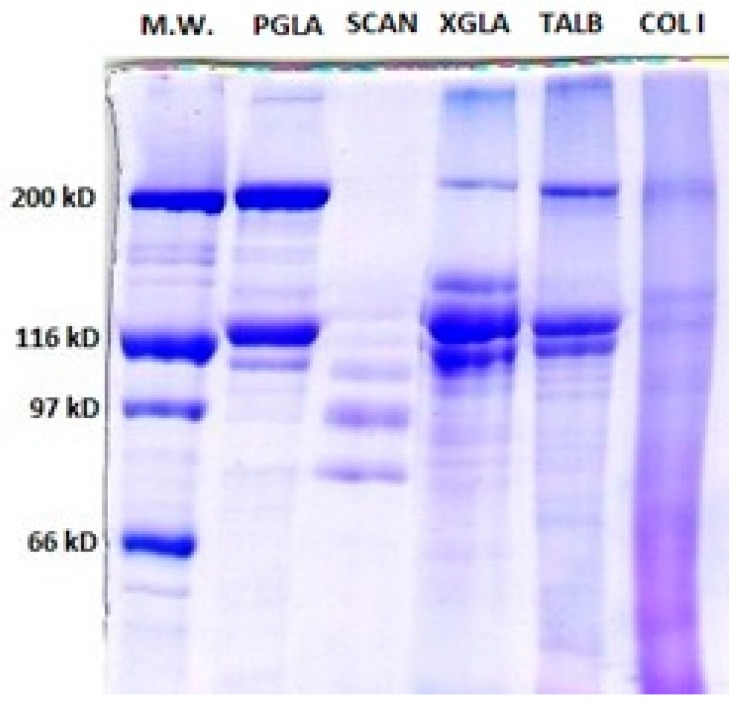
7% Sodium Dodecyl Sulfate-Polyacrylamide Gel Electrophoresis (SDS-PAGE) showing Pepsin soluble collagen (PSC) from *Prionace glauca* (PGLA), *Scyliorhinus canicula* (SCAN), *Thunnus albacares* (TALB) and *Xiphias gladius* (XGLA). M.W: Molecular Weight Standards. Col I: standard collagen type I from mammal.

Figure 1 shows the PSC electrophoretic patterns of the analysed species. The PSC SDS-PAGE pattern from PGLA and TALB were more similar to the type I collagen pattern where two identical *α*_1_-chains (120 kDa), one *α*_2_-chain (110 kDa), and one *β* dimer band of about 200 kDa can be observed [16,31]. The molecular weight data obtained for *α* and *β* chains of PSC from TALB are similar to those previously published for the same species [23,32]. The cross-linking rate of collagen has been reported to be low; which might explain why highly cross-linked components (*γ*-component) in PGLA, TALB, and XGLA are shown only as a faint bands in Figure 1 [33,34]. This result indicates that pepsin was able to hydrolyse the cross-links in the telopeptide region without damaging the integrity of the triple-helix.

PSC from SCAN was characterized by a high susceptibility to pepsin hydrolysis, as revealed by the fact that neither dimer nor trimer could be observed in SDS-PAGE, and also by the presence of several weak *α* subunits lower than 110 kDa, which could be products of enzymatic hydrolysis of collagen components (Figure 1). In fact, previous publications have shown that *β* and *γ*-components were present in acid soluble collagen from SCAN skin [19].

In the electrophoretic pattern of XGLA, one intermediate band was observed between the *β* and *α* component with an approximate molecular weight of about 150 kDa. The presence of similar components have also been reported for PSC from different species, suggesting either an incomplete hydrolysis of *β* dimers, or the presence of a mixture of different collagens [35,36].

##### Amino Acid Content

Table 3 shows the amino acid composition of the PSC of the four studied species and also that from calf skin (data obtained from Zhang et al. [21]). To our knowledge, amino acid composition has never previously been reported for PSC collagen of these species except for TALB [32]. Although, Glycine was the most abundant amino acid in all the species studied, yet did not represent one third of the total amino acid residues as expected [19,20]. Similar results have been previously reported in PSC obtained from yellowfin tuna skin [32] and squid skin collagen [7]. This result might be explained due to the presence of telopeptide fractions in which the repetitive occurrence of glycine every three amino acid is absent [30].

The lower imino acid content found in SCAN PSC, contributes to the low stability of the triple helix structure [35], which is a result that is in agreement with the SDS profiles shown above, indicating the higher susceptibility of this species to the action of pepsin.

##### Determination of Denaturation Temperature

DSC analyses of lyophilized PSC were performed. Calf skin type I collagen was used for comparison purposes. Denaturation temperatures for PGLA, SCAN, TALB, and XGLA PSCs were 33 °C, 23.6 °C, 30.6 °C, and 31.4 °C respectively, which are similar to those found in literature for other PSC in different marine organisms: paper nautilus [37], striped catfish [38], bighead carp [35], or blueshark [39]. Denaturation temperatures of PSC in all species were lower than that of collagen type I of calf skin (T_d_ = 40 °C). Among the four species studied, the lower denaturation temperature was found in SCAN PSC. These results agree with the lower imino acid content (hydroxyproline and proline) found in the collagen obtained from this species. Thermal stability of collagen is related to the restriction of the secondary structure imposed by the pyrrolidine rings of proline and hydroxyproline, contributing to the strength of the triple helix [20,40]. Sotelo et al. [19] have found a higher denaturation temperature for ASC obtained from small-spotted catshark skin, suggesting the influence of pepsin cross-link cleavage on lower thermal stability found in PSC. Similar results were obtained for ASC and PSC from the skin of brownbanded bamboo shark [32].

### 2.3. Enzymatic Hydrolysis of PSC

#### 2.3.1. Degree of Hydrolysis

Hydrolysis curves were similar to others previously reported for different marine skin proteins [41,42]. The hydrolysis degree (DH) (average values ±SD) calculated using the pH-STAT method were 16.52 ± 3.74%, 15.80 ± 0.99%, 11.49 ± 1.5%, and 12.56 ± 1.79% for PGLA, SCAN, TALB, and XGLA, respectively. Enzymatic proteolysis and the resulting degree of hydrolysis are key parameters influencing peptide length and other related characteristics such as solubility, nutritional, functional, or sensory properties [7,9].

#### 2.3.2. Antioxidant Activities in Hydrolysates

Table 4 shows data of antioxidant analysis in collagen unfractionated hydrolysates (H) and 3kDa ultrafiltration fractions: retentates (R) and permeates (P). The antioxidant capacities were evaluated using 3 methods, including two based on free radical scavenging capacity, that is, DPPH and ABTS, and one based on the inhibition of lipid peroxidation, determined by the *β*-carotene assay.

The precise mechanism explaining the antioxidant activity of peptides has not been entirely elucidated, however several authors suggested the influence of hydrolysis degree [14,15]. As it was expected, hydrolysate (H) fractions, determined with DPPH and ABTS exhibited lower values of antioxidant activity in the hydrolysate with the highest hydrolysis degree (PGLA). However, the highest values of antioxidant activity were found in XGLA which showed a higher hydrolysis degree than TALB, suggesting the influence not only of the hydrolysis degree but also to the presence of some amino acids such as cysteine which may interact with free radicals by their SH groups [14,43,44,45]. Thus, while XGLA hydrolysate presented the highest values of cysteine content (53.03/1000 residues), PGLA hydrolysate showed a low cysteine content (8.93/1000 residues) (Table 5). On the other hand, the *β*-carotene method showed highest antioxidant capacity with those hydrolysates with the highest DH (SCAN and PGLA), while those with the lowest DH showed also the lowest antioxidant capacity (Table 4).

**Figure 2:**
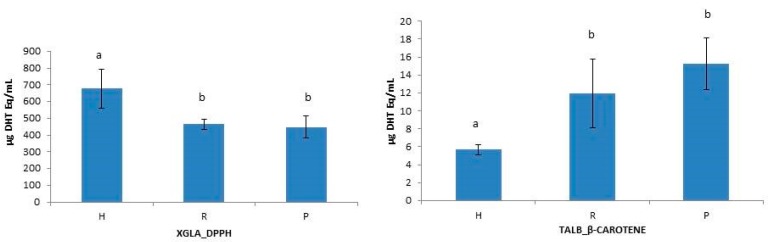
Intraspecific differences between hydrolysate (H), retentate (R) and permeate (P) in XGLA analyzed by DPPH method and in TALB analyzed by *β*-Carotene method. Different letters indicate significant differences among means (*p* ≤ 0.05).

**Figure 3:**
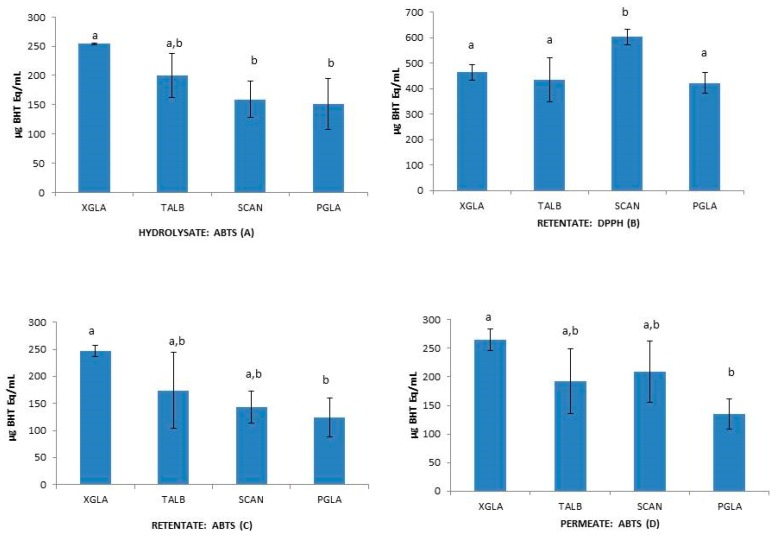
Interspecies differences in hydrolysate fraction using ABTS (**A**); in retentate fraction using DPPH (**B**) and ABTS (**C**); in permeate fraction using ABTS (**D**). Different letters indicate significant differences among means (*p* ≤ 0.05).

To test the influence of molecular size reduction of peptides on the functional properties of collagen hydrolysates [10,14,46], the antioxidant capacity of unfractionated hydrolysates (H), retentates (R) and permeates (P) were statistically analyzed. One-way ANOVA analysis of data revealed some significant intraspecific differences between H, R, and P when using *β*-Carotene and DPPH methods (Figure 2) and also showed interspecies differences between H, R, and P when using DPPH and ABTS methods (Figure 3). The unfractionated hydrolysate (H) of XGLA showed significant higher value (*p* ≤ 0.05) of antioxidant activity determined with DPPH compared to retentate or permeate fractions (Figure 2). Significant differences were also observed in TALB, when data from the *β*-Carotene method were analyzed, between unfractionated hydrolysate and the other two fractions (R and P). Interspecies significant differences of hydrolysates, retentates, and permeates are presented in Figure 3 (*p* ≤ 0.05). Figure 3A shows the differences found for unfractionated hydrolysates with ABTS; XGLA showed the highest antioxidant activity whereas SCAN and PGLA were the lowest. However, unfractionated hydrolysates did not show significant differences between species when the antioxidant activity was determined with DPPH or the *β*-Carotene method (data not shown). In Figure 3B, it can be also observed that the retentate fraction of SCAN presented the highest activity compared to other three species when DPPH was used, while ABTS data (Figure 3C) showed significant differences in retentate fractions only between XGLA and PGLA (lowest). Regarding permeate fractions (Figure 3D), significant differences were observed only between XGLA and PGLA when ABTS data were analyzed.

Significant differences (*p* ≤ 0.05) were observed between the antioxidant capacity of unfractionated hydrolysates of teleost (XGLA and TALB) and chondrychtyes (PGLA and SCAN) with the *β*-carotene assay. Thus, the two teleost species XGLA and TALB showed lower antioxidant capacity than chondrychtyes, results that might be in relation with the higher content of hydrophilic amino acids (Asp, Ser, Gly, His, Arg, Thr, and Cys) in chondrychtyes hydrolysates compared to teleost (Table 5).This result agree with other studies suggesting differences on the antioxidant defense system between elasmobranchs and teleosts, due to different evolutionary rates and also due to different physical activity, nutrient intake and environment in which each species develops [47].

In summary, antioxidant capacity results suggest that there is not a unique factor responsible for this antioxidant capacity of hydrolysates, which seems to be influenced by the species which is being studied, the type and length of the peptides present in the sample and the methodology employed to determine the antioxidant activity.

#### 2.3.3. Amino Acid Content

Table 5 shows the amino acid content of unfractionated collagen hydrolysates. Besides the influence of amino acid composition and other factors on antioxidant activity (discussed above), it is also of importance to highlight the increase in Cystine content in hydrolysates, in comparison to non-hydrolyzed collagen (PSC). These variations might be explained because the alkaline pH achieved during hydrolysis promotes reoxidation of cysteine residues to generate the original disulfide bond [48]. The higher Cystine content found in TALB and XGLA hydrolysates is therefore related to the low collagen yield obtained for those skins (Section 2.2.1). As it was previously reported, the positive correlation between high disulfide bond content and low extraction yields is because of a higher stabilization of supramolecular assemblies [49]. The higher content of methionine in SCAN hydrolysates compared to the other species is also noteworthy.

## 3. Experimental Section

### 3.1. Raw Material

Fresh skin of the small-spotted catshark was obtained by a local fishing fleet, while frozen skin of blue shark, swordfish, and yellowfin tuna was provided by a Lumar S.L industry (Galicia, Spain) and stored at −20 °C until used. Fins, fat, and muscle residues were removed from skins, then skin was cut into small pieces (0.5 cm × 0.5 cm) and mixed thoroughly. The skin pieces of each species were divided into three batches which were kept frozen at −20 °C until collagen extraction.

Identification of fish species was performed by DNA analysis, following the methodology of Blanco et al. [50].

#### 3.1.1. Proximate Composition

Skin was analyzed for crude protein content by Kjeldhal method [51] in a DigiPREP HT digestor (SCP Science, Quebec, QC, Canada) and a TitroLine easy titration unit (SCHOTT, Mainz, Germany). Lipid content was determined by Bligh and Dyer [52]. Moisture was determined after heating the sample overnight at 105 °C and ash content was determined after heating the sample overnight at 600 °C. The conversion factor used for calculating the protein content from Kjeldahl nitrogen data was 5.4 as collagen, the main protein present in skin, contains approximately 18.7% nitrogen [53,54].

#### 3.1.2. Hydroxyproline Content

30 mg of dried grinded skin was introduced in hydrolysis microwaves tubes and 4 mL of 6 M HCl were added. Hydrolysis was performed in a microwave (speed wave MWS-2) (Berghof GmbH, Eningen, Germany) at a 150 °C for 90 min at 70% power. Once the hydrolysis step finished, samples were allowed to cool down to room temperature and were made up to a known volume with 6 M HCl. 400 µL of this solution were transferred to glass vials and left to dry in a vacuum desiccator at 60 °C in the presence of solid NaOH, after drawing air for 3 days. The resulting dry matter was suspended in 8 mL of buffer (0.13 M citric acid, 0.75% glacial acetic acid, 0.6 M sodium acetate, 0.15 M sodium hydroxide and 20.13% *n*-propanol, pH was adjusted to 6.5 with 0.2 M NaOH and volume was brought to 660 mL with distilled water).

Hydroxyproline primary standard was prepared by dissolving 50 mg of hydroxyproline (Sigma-Aldrich, St. Louis, MO, USA) in 100 mL of buffer. From this primary standard a calibration curve of hydroxyproline, ranging from 0.5 µg/mL up to 10 µg/mL, was prepared. Chloramine-T reagent was freshly prepared just before using it (0.05 M Chloramine in distilled water). 3 mL of either samples or standards were placed in a tube and 1.5 mL of Chloramine-T reagent was added, the mixture was allowed to react for 25 min. Upon completion of that time, chromogenic reagent (15 g of *p*-dimethyl-amino-benzaldehyde, 60 mL of *n*-propanol, 26 mL of 70% perchloric acid were made up to a volume of 100 mL with distilled water) was added and tubes introduced in a water bath at 60 °C for 15 min. Samples were left to cool to room temperature and after, absorbance was read at 550 nm in a Beckman UV-VIS spectrophotometer (Beckman-Coulter, Brea, CA, USA).

### 3.2. Extraction of Pepsin Soluble Collagen (PSC) from Skin

**Figure 4:**
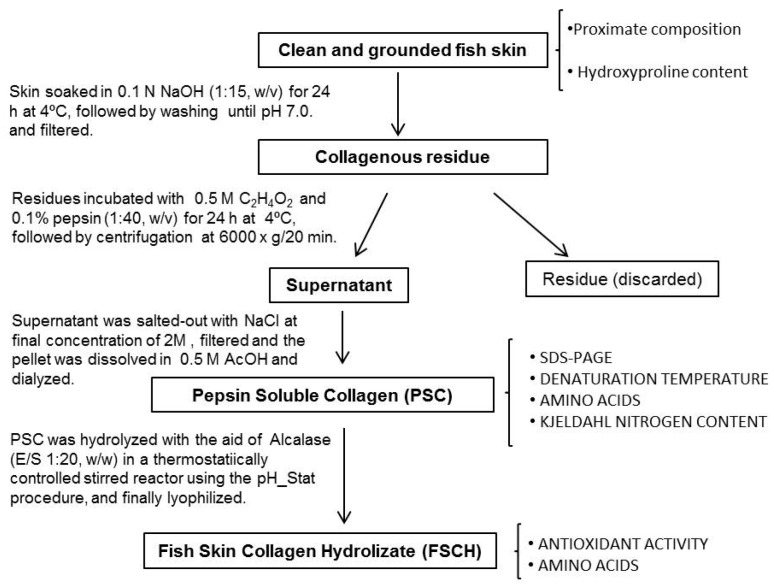
Scheme for the recovery of pepsin soluble collagen (PSC), preparation of the hydrolysate and analytical determinations.

Collagen from skin was extracted according to the methodology of Liu et al. [35] with minor modifications (Figure 4). All procedures were performed at 4 °C. Skin pieces of blue shark and small-spotted-catshark were first treated with 0.1 N NaOH (1:15, *w*/*v*) and stirred for 24 h. Then, skins were washed with cold distilled water until a neutral pH was found, and skin residues were extracted with 0.5 M acetic acid containing 0.1% (*w*/*v*) pepsin (0.5 U/mg; Acros Organics, Janssen Pharmaceuticalaan 3a, Geel, Belgium), at a sample solution ratio of 1:40 (*w*/*v*) for 24 h. Suspension was centrifuged at 6000× *g* for 20 min, the residue discarded and the supernatant was salted-out by adding NaCl (final concentration of 2 M). The precipitate was dissolved in 0.5 M acetic acid and dialyzed against water using 12,000 Da cut-off membranes for 3 days. Aliquots were obtained and freeze-dried for analysis of Kjeldahl nitrogen, amino acid content, denaturation temperature, and electrophoresis. The remaining liquid volume of dialyzed PSC was stored frozen at −20 °C until used for hydrolysis.

The procedure used for swordfish and yellowfin tuna skin was slightly different than the one employed with sharks. Higher fat content in both swordfish and tuna skin required that after alkaline treatment and before the acid pepsin extraction, samples were soaked in 10% butyl alcohol for 24 h to remove any remaining fat at a sample/solid ratio of 1:10 (*w*/*v*), and then washed until neutral pH. Also thre time for pepsin extraction of these skins was increased up to 3 days.

PSC yields were calculated using Kjeldahl nitrogen values (data not shown) in the collagen solution considering that collagen contains approximately 18.7% of nitrogen [53,54].

### 3.3. Characterization of Pepsin Soluble Collagen (PSC) from Skin

#### 3.3.1. Polyacrylamide Gel Electrophoresis

PSC samples for Sodium Dodecyl Sulfate-Polyacrylamide Gel Electrophoresis (SDS-PAGE) were prepared according to methodology reported by Sotelo et al. [19]. Molecular weights of PSC subunits were estimated using high range molecular weight standards (BIO-RAD): Myosin (200 kDa); *β*-Galactosidase (116 kDa); phosphorylase B (97 kDa) and analyzing the gel with the software Quantity One (BIO-RAD).

#### 3.3.2. Differential Scanning Calorimetry

Freeze-dried PSC samples were solubilized in 50 mM acetic acid (1 mg of freeze-dried sample/mL). Thermostability of PSC solutions was measured in a DSC III microcalorimeter (Setaram, France) by differential scanning calorimetry (DSC). The samples were weighed accurately in a Mettler AE-240 balance, introduced into the calorimeter at 283.15°K and left for one hour to stabilize. Afterwards, temperature increase was set to 1°K/min up to 343°K. The denaturation temperature was calculated by difference with the apparent specific heat of ultrapure water.

#### 3.3.3. Nitrogen Content

PSC was analyzed in terms of nitrogen content by Kjeldahl method described in Section 3.1.1 considering a 5.4 factor to obtain the collagen content.

#### 3.3.4. Amino Acid Composition

100 mg of lyophilized PSC samples were hydrolyzed using 6 N hydrochloric acid under vacuum pressure at 110 °C for 24 h. HPLC-fluorescence determination of amino acids, using AccQ-Tag Amino acid analysis column (Waters Co., Milford, MA, USA), was carried out after derivatization using the AccQ-Tag Chemistry kit (Waters-WAT052875).

### 3.4. Enzymatic Hydrolysis of Pepsin Soluble Collagen

Enzymatic hydrolysis was carried out according to the methodology of Liu et al. [35] with minor modifications. Prior to the hydrolysis process, the selected volume of each PSC collagen batch was thawed. Hydrolysates were prepared in a stirred and thermostated reactor connected to a pH electrode and a temperature probe, using the pH-Stat procedure, as described by Adler-Nissen [55]. Temperature and pH were recorded by a visual display at all time. Food-grade Alcalase (2.4 AU-A/g) provided by Novo Nordisk (Bagsvaerd, Denmark) was used for the hydrolysis. The 2 L of thawed PSC were introduced in the reactor and heated up to 55 °C (Alcalase optimum temperature), pH was adjusted to pH 8.0 with 1 N NaOH and maintained constant during the hydrolysis reaction by automatically adding 1 N NaOH. Hydrolysis started with the addition of enzyme (enzyme/protein ratio of 1:20 *w*/*w*). The hydrolysis reaction was allowed to continue for 3 h under constant stirring. At the end of hydrolysis, the enzyme was inactivated by heating at 90 °C for 5 min. The resulting hydrolysates were freeze-dried and kept frozen at −20 °C until characterization analysis.

#### Degree of Hydrolysis

Degree of hydrolysis (DH) was obtained according to the following expression [55,56] where DH is the percent ratio between the total number of peptide bonds cleaved and the total number of peptide bonds in the initial protein.

(1) $$DH\left(\%\right)=\frac{B\times N_{b}}{\alpha \times M_{p}\times h_{tot}}$$

where *B* is the volume (mL) of 1 M NaOH consumed during hydrolysis; *N_b_* is the normality of NaOH; *M_p_* is the mass (g) of initial protein (nitrogen × 5.4); *h_tot_* is the total number of peptide bonds available for proteolytic hydrolysis, and *α* is the average degree of dissociation of the amino groups in the protein substrate and was calculated as follows:

(2) $$\alpha =\frac{10^{pH-pK}}{1+10^{pH-pK}}$$

The *pK* value dependent on the temperature of hydrolysis was calculated according to the following expression, where *T* is the temperature (*K*):

(3) $$pK=\left[7.8+\frac{298-T}{298T}\right]\times 2400$$

*h_tot_* was calculated considering a mean molecular weight of amino acids around 125 g/mol [57], and total content of amino acid in each PSC obtained from different species (PGLA: 78.4 g/100 g; SCAN: 96.02 g/100 g; TALB: 92.75 g/100 g; XGLA: 80.84 g/100 g). *h_tot_* of PSC collagen were 6.8 meq/g protein, 8.3 meq/g protein, 8.06 meq/g protein and 7.02 meq/g protein for PGLA, SCAN, TALB, and XGLA respectively.

### 3.5. Antioxidant Capacity of Pepsin Soluble Collagen Hydrolysates

#### 3.5.1. Ultrafiltration

To test the influence of molecular weight on antioxidant capacity, four grams of freeze-dried hydrolysates were dissolved in distilled water (1%) and ultrafiltrated in two steps using ultrafiltration centrifugal devices (Amicon Ultra-15 Unit) (Merck Millipore, Billerica, MA, USA) with molecular weight cut-off of 10 kDa and 3 kDa. After this process, fractions containing peptides with molecular weight between 10,000 Da and 3000 Da (retentate fraction) and fractions containing peptides below 3000 Da (permeate fraction) were then freeze-dried and stored at −20 °C until subjected to antioxidant capacity analysis.

#### 3.5.2. Antioxidant Activity Determinations

##### *β*-Carotene Bleaching Method

The *β*-carotene bleaching assay was performed according to Prieto et al. 2012 [58] with a microplate spectrophotometer. Reactions were performed by combining in each well of a 96-well microplate, 25 μL of antioxidant (butyl hydroxytoluene (BHT) at 0–22.7 μM or hydrolysate samples) with 125 μL of the *β*-carotene/linoleic emulsion. The microplate spectrophotometer (Multiskan Spectrum Microplate Spectrophotometer) (Thermo Fisher Scientific, Waltham, MA, USA) was programmed to record the absorbance at 470 nm and 45 °C every three minutes during a period of 200 min with agitation at 660 cycles/min (1 mm amplitude).

##### 1,1-Diphenyl-2-Picryhydrazyl (DPPH) Radical-Scavenging Capacity

The antioxidant activity as radical-scavenging capacity was assessed with DPPH as a free radical, using an adaptation to the microplate of the method described by Brand-Williams et al. [59,60]. The decrease in the absorbance of hydrolysates and the BHT control (0–108 µM) was followed at 515 nm every 3 min during 200 min at 30 °C.

##### ABTS Bleaching Method

The ABTS (2,2′-azinobis-(3-ethyl-benzothiazoline-6-sulphonic acid) radical scavenging activities were assessed according the protocol developed by Prieto et al. [60]. The absorbance at 414 nm and 30 °C (maintaining continuous agitation) of samples and BHT (0–9.1 µM) were measured each 3 min in the microplate reader.

In all methods, the kinetics of reaction were performed in triplicate following the methodology of Amado et al. (2016) [61].

#### 3.5.3. Amino Acid Composition

Hydrolysates were analyzed for amino acid content following the methodology described in Section 3.3.3.

#### 3.5.4. Statistical Analysis

Interspecific and intraspecific differences regarding antioxidant capacity between unfractionated hydrolysates (H) and 3 kDa MWCO ultrafiltrated fractions: permeates (P) and retentates (R) were tested by one-way analysis of variance (ANOVA). It was applied to a Post hoc comparison test. Significance levels were set at *p* ≤ 0.05. Statistical tests were performed with IBM SPSS 23 (IBM Corporation, Armonk, NY, USA).

## Figures and Tables

**Table 1:** Chemical composition of fish skins from the four species used for the study. Values, expressed in a wet basis, are means of 3 determinations ± standard deviation (Protein = *N* × 5.4).

Species	Composition (%)			
	Moisture	Protein	Lipids	Ash
PGLA	76.03 ± 0.83	20.14 ± 0.97	0.24 ± 0.03	4.24 ± 0.24
SCAN	61.5 ± 0.79	22.09 ± 0.96	0.36 ± 0.01	14.01 ± 0.5
XGLA	42.87 ± 0.54	16.28 ± 2.21	30.53 ± 1.99	2.49 ± 0.21
TALB	62.57 ± 2.4	26.96 ± 2.04	3.22 ± 0.72	0.67 ± 0.14

**Table 2:** Hydroxyproline (OHPro) content in skin (g OHPro/100 g skin), collagen content calculated from the hydroxiproline values, and yield of PSC_1_ (g collagen/100 g skin), and PSC_2_ (g collagen/100 g collagen of the skin). The average values (±SD) expressed in a wet weight basis are means of three replicates.

	Hydroxyproline Content in Skin (%)	Collagen Content (%)	PSC_1_ Yield (%)	PSC_2_ Yield (%)
PGLA	1.23 ± 0.11	9.84 ± 0.88	5.87 ± 0.49	61.17 ± 5.15
SCAN	1.85 ± 0.14	14.8 ± 1.14	4.89 ± 0.85	33.00 ± 5.25
XGLA	1.08 ± 0.16	8.64 ± 1.28	2.59 ± 0.22	31.33 ± 5.55
TALB	2.69 ± 0.26	21.53 ± 2.09	2.97 ± 0.98	14.16 ± 6.14

**Table 3:** Amino acid composition of PSC of PGLA, SCAN, TALB and XGLA (residues/1000). Data from calf skin collagen is also included [21]. Imino acids includes proline and hydroxyproline.

Amino Acid	PSC				CALF
	PGLA	SCAN	TALB	XGLA	
Hydroxyproline	84.62 ± 0.98	88.28 ± 0.62	87.38 ± 0.60	76.55 ± 0.87	94
Aspartic acid	46.58 ± 0.42	52.16 ± 0.43	55.40 ± 0.54	61.32 ± 0.46	45
Serine	35.98 ± 0.42	54.02 ± 0.14	35.53 ± 0.25	39.89 ± 0.74	33
Gultamic acid	92.02 ± 1.00	92.10 ± 0.47	97.89 ± 0.43	94.64 ± 0.96	75
Glycine	214.80 ± 2.92	234.69 ± 1.36	217.22 ± 1.32	210.20 ± 3.22	330
Histidine	15.80 ± 0.20	17.35 ± 0.10	12.70 ± 0.05	15.67 ± 0.34	5
Arginine	111.50 ± 1.09	91.26 ± 1.08	92.16 ± 2.97	89.54 ± 2.26	50
Threonine	33.59 ± 0.16	33.41 ± 0.44	40.00 ± 1.81	42.89 ± 1.60	18
Alanine	108.57 ±0.87	89.79 ± 0.97	111.78 ± 2.58	105.20 ± 2.39	119
Proline	107.68 ± 0.76	95.22 ± 0.29	114.86 ± 0.45	121.89 ± 1.30	121
Cystine	0.88 ± 0.01	0.31 ± 0.00	0.07 ± 0.00	0.61 ± 0.01	0
Tyrosine	3.39 ± 0.05	1.36 ± 0.00	4.42 ± 0.07	6.45 ± 0.15	3
Valine	27.77 ± 0.39	34.13 ± 0.12	25.64 ± 0.15	26.95 ± 0.40	21
Methionine	13.51 ± 0.33	14.06 ± 0.20	6.29 ± 0.13	3.53 ± 0.15	6
Lysine	33.48 ± 0.36	37.78 ± 0.13	35.37 ± 0.23	31.52 ± 0.43	26
Isoleucine	24.62 ± 0.30	18.29 ± 0.02	14.26 ± 0.15	20.47 ± 0.38	11
Leucine	25.97 ± 0.36	27.30 ± 0.07	28.28 ± 0.21	31.19 ± 0.68	23
Phenylalanine	19.25 ± 0.22	18.49 ± 0.01	20.75 ± 0.15	21.50 ± 0.47	3
Iminoacids	192.3	183.5	202.24	198.44	215
% hydroxylation of proline	44.00	48.10	43.20	38.57	44

**Table 4:** Antioxidant activities (Mean ± SD) of collagen unfractionated hydrolysates (H), retentates (R) and permeates (P) quantified by means of three methods (DPPH, ABTS, and *β*-carotene) and calculated as equivalents (in µg) of BHT per mL of hydrolysate.

Species	Fraction	DPPH (mg BHT Eq/mL)	ABTS (mg BHT q/mL)	*β*-Carotene (mg BHT Eq/mL)
XGLA	H	677.20 ± 114.42	253.77 ± 1.85	7.59 ± 1.93
TALB	H	578.87 ± 57.81	199.57 ± 37.54	5.67 ± 0.61
SCAN	H	494.17 ± 210.3	159.17 ± 30.78	20.86 ± 3.53
PGLA	H	405.30 ± 9.89	151.20 ± 43.49	15.26 ± 5.02
XGLA	R	465.63 ± 30.47	247.27 ± 10.70	5.91 ± 1.04
TALB	R	435.97 ± 85.54	174.10 ± 70.05	11.94 ± 3.86
SCAN	R	603.40 ± 30.88	143.57 ± 29.80	7.38 ± 11.69
PGLA	R	422.97 ± 41.32	124.90 ± 35.76	19.18 ± 1.92
XGLA	P	448.0 ± 66.45	264.87 ± 18.86	8.08 ± 0.33
TALB	P	457.67 ± 95.61	192.83 ± 56.66	15.26 ± 2.91
SCAN	P	601.70 ± 175.33	209.70 ± 53.71	12.40 ± 9.14
PGLA	P	416.03 ± 18.88	134.87 ± 26.76	17.03 ± 2.64

**Table 5:** Amino acid composition of collagen hydrolysates of four species (residues/1000). Imino acids includes proline and hydroxyproline.

Amino Acid	HYDROLYSATES			
	PGLA	SCAN	TALB	XGLA
Hydroxyproline	84.65 ± 0.80	87.50 ± 1.22	86.97 ± 0.54	75.15 ± 0.36
Aspartic acid	48.56 ± 0.45	53.33 ± 0.77	53.08 ± 0.24	59.39 ± 0.34
Serine	36.39 ± 0.34	52.45 ± 0.65	34.81 ± 0.20	38.83 ± 0.19
Gultamic acid	92.49 ± 0.89	90.97 ± 1.27	90.69 ± 0.42	92.02 ± 0.43
Glycine	230.71 ± 2.10	227.17 ± 2.96	215.82 ± 0.66	211.01 ± 1.06
Histidine	16.53 ± 0.13	16.49 ± 0.18	11. 18 ± 0.12	14.91 ± 0.03
Arginine	93.64 ± 0.98	93.00 ± 1.08	90.92 ± 0.65	76.46 ± 0.16
Threonine	27.99 ± 0.32	36.62 ± 0.59	40.00 ± .035	39.00 ± 0.26
Alanine	105.81 ±1.11	93.50 ± 1.27	108.72 ± 0.74	97.97 ± 0.62
Proline	106.47 ± 1.14	89.31 ± 1.26	100.22 ± 0.77	99.87 ± 0.61
Cystine	8.93 ±0.16	8.29 ± 0.33	31.91 ± 0.33	53.03 ± 0.16
Tyrosine	2.17 ± 0.01	1.68 ± 0.02	1.84 ± 0.02	2.24 ± 0.00
Valine	27.84 ± 0.28	34.12 ± 0.42	26.17 ± 0.17	27.61 ± 0.12
Methionine	13.68 ± 0.15	17.06 ± 0.26	15.19 ± 0.24	12.39 ± 0.09
Lysine	34.16 ± 0.32	37.55 ± 0.48	33.88 ± 0.14	32.70 ± 0.17
Isoleucine	24.65 ± 0.26	17.45 ± 0.20	13.05 ± 0.10	19.15 ± 0.09
Leucine	26.11 ± 0.25	25.95 ± 0.27	26.20 ± 0.13	28.58 ± 0.07
Phenylalanine	19.23 ± 0.19	17.56 ± 0.17	19.34 ± 0.10	19.67 ± 0.04
Iminoacids	191.12	176.81	187.19	175.02
% hydroxylation of prol	44.29	49.48	46.45	42.93
